# Synchronous Breast and Colorectal Malignant Tumors—A Systematic Review

**DOI:** 10.3390/life14081008

**Published:** 2024-08-13

**Authors:** Cristian Iorga, Cristina Raluca Iorga, Alexandru Grigorescu, Iustinian Bengulescu, Traian Constantin, Victor Strambu

**Affiliations:** 1 Faculty of Medicine, “Carol Davila” University of Medicine and Pharmacy, 050474 Bucharest, Romania; cris.iorga@yahoo.com (C.I.); alexgrigorescu2004@yahoo.com (A.G.); iustinian.bengulescu@umfcd.ro (I.B.); traianc29@yahoo.com (T.C.); drstrambu@gmail.com (V.S.); 2 Surgery Clinic, “Dr. Carol Davila” Clinical Nephrology Hospital, 010731 Bucharest, Romania; 3 Oncology Department, “Dr. Carol Davila” Clinical Nephrology Hospital, 010731 Bucharest, Romania; 4 Department of Urology, “Prof. Dr. Th. Burghele” Hospital, 050652 Bucharest, Romania

**Keywords:** synchronous, breast tumors, colorectal tumors

## Abstract

The incidence of breast and colorectal cancers is well established in studies, but the synchronous occurrence of the two types of tumors is a rarity. In general, they are discovered during screening investigations following the diagnosis of an initial tumor. Objective: Our aim is to describe the main diagnostic and therapeutic challenges for synchronous breast and colorectal tumors. Materials and methods: We performed a systematic review of the literature for cases or case series, using established keywords (synchronous breast and colon tumor and synonyms) for the period of 1970–2023. Five reviewers independently screened the literature, extracted data, and assessed the quality of the included studies. The results were processed according to the PRISMA 2020 guidelines. Results: A total of 15 cases were included in the study, including 2 males (age 50 and 57) and 13 females (median age 60, with range from 40 to 79). In a vast majority of the cases, the diagnosis of synchronous tumor was prompted by the first tumor’s workup. The first diagnosed tumor was colorectal in nine cases and a breast tumor in six cases. The most common histopathological type of breast tumor was invasive ductal carcinoma, and the colon tumors were exclusively adenocarcinomas. All cases had a surgical indication for both breast and colorectal tumor, except one case, in which the breast tumor had multiple metastasis. In four cases, the surgery was performed concomitantly (colectomy and mastectomy). In three cases, surgery was initially carried out for the breast tumor, followed by colon surgery. Oncological treatment was indicated depending on the tumor stage. Conclusions: For the treatment of synchronous tumors, the Tumor Board (T.B) decision is mandatory and must be personalized for each patient. Developing new methods of treatment and investigation may play an important role in the future for understanding synchronous tumor development, incidence, and outcome.

## 1. Introduction

Breast cancer is the most common malignancy in women worldwide and is responsible for approximately 40,000 deaths/year in the USA. For the year 2024, the National Cancer Institute estimates 15.5% new cases and a mortality of 6.9%, with a relative survival rate at 5 years of 91.2%. The median age at onset is 63 years [1,2,3,4].

Colon cancer is the third most common form of neoplasia worldwide and is the second most common form of neoplasia in women. For the year 2024, the National Cancer Institute estimates 7.6% new cases and a mortality of 8.7%, with a relative survival rate at 5 years of 65%. The median age of onset is 67 years [4,5,6,7].

Synchronous tumors are categorized as multiple primary malignant tumors (MPMTs) and are defined as tumors (two or more) with different topographic locations and different histologic characteristics that are diagnosed at the same time or within 6 months of the first tumor and are not metastatic from each other. Although they were initially considered rare entities, more and more studies have recently shown that the incidence of MPMTs is increasing [8,9,10].

The first descriptions of MPMTs date back to 1889, communicated by Billroth at al., and the current incidence is between 0.73 and 11.7%; synchronous tumors of the breast and colon have an incidence of 3.85% [11,12,13].

A literature review (De Luca et al., 2019) found that the most common MPMT associations of breast cancer were, in descending order of frequency, as follows: skin cancer (especially melanoma); gastrointestinal cancer (especially colorectal cancer); and gynecological cancer (the most common association being with ovarian cancer). Less frequent associations were found between breast cancer and sarcoma and between lung cancer and urological tumors (especially bladder). The association with thyroid cancer is inverse in that women who have developed thyroid cancer have a much higher risk of developing breast cancer [9].

The mechanisms by which these associations occur are intensively discussed in the literature and are still unclear. Past studies have indicated that the following factors are involved: BRCA and Lynch syndrome gene mutation, lifestyle (tobacco and alcohol abuse, lack of physical activity), age, decreased immunity, and, last but not least, oncologic treatment (radiation and chemotherapy). Patients with cancer had 1.29 times the risk of developing a new malignancy compared with those that were never diagnosed with cancer [9].

Up to 35% of patients with colorectal cancer have a family history of the disease, suggesting a hereditary predisposition, while only 10% of breast cancer diagnoses are familial in nature. It has been proven that there is correlation between family history and synchronous tumors [14].

BRCA 1 and BRCA 2 (breast cancer antigenes) are inherited genes and produce proteins that help repair damaged DNA. The risk of developing breast and ovarian cancer is about four times higher in carriers of this mutation (45–72% versus 16% in the general population) [15,16].

Lynch syndrome is an autosomal dominant inherited mutation of genes regulating DNA mismatch repair (MLH1, MSH2, MSH6, and PMS2) [17].

There is also an increased risk for other types of cancers as a result of this mutation, particularly for endometrial, gastric, ovarian, biliary, urinary tract, small bowel, brain, and pancreatic cancers. In Lynch syndrome, mutations in mismatch repair allow for the proliferation of aberrant microsatellites within tumor suppressor genes, resulting in cancerous growth.

The lifetime risk for colorectal cancer is 80% and up to 60% for endometrial cancer [18].

However, many MPMTs still remain incidental findings, as they are sporadic cases without a mechanism of occurrence [19,20].

This study aimed to describe the main diagnostic and therapeutic challenges for patients diagnosed with synchronous breast and colorectal tumors.

## 2. Materials and Methods

### 2.1. Search Strategy

We searched databases (Pub Med, Pub Med Central, Google Scholar, Cochrane, and Registries) on 25 October 2023, using the following terms: (Synchronous OR Concomitant) AND (Cancer OR Carcinoma OR Neoplasia) AND (Colorectal OR Colon OR Rectal) AND (Breast). PRISMA guidelines were followed during the data extraction, analysis, and reporting [21]. Articles published in the English language between 1970 and 2023 were selected.

### 2.2. Study Selection, Data Extraction, and Quality Assessment

Eligibility criteria for the systematic review were as follows: case reports referring to synchronous breast and colorectal tumors, available in English, for which the integral text was found. We excluded articles referring to other types of synchronous tumors (or with more than two synchronous tumors other than breast and colorectal); cases with prior diagnosis or treatment for another neoplasm; cases for which the full text was not available; and articles available in a language other than English.

Abstracts and selected titles were initially screened for duplicates and for compliance with the eligibility criteria by three people (C.R.I, C.I, T.C) who worked independently. In case of doubt, another reviewer (V.S) would decide. After this first step, the remaining articles were fully reviewed by two other people (I.B, A.G) who analyzed whether they met the eligibility criteria and corresponded to the aim of the study. Manual searches were carried out if the articles appeared to be relevant.

From the articles included in the study, the following data were extracted: the author’s name and year of publication, demographic data (age, sex, risk factors, history), tumor data (which tumor was diagnosed first, tumor stage, histopathology), treatment data (therapeutic sequence (surgical/oncologic), treatment and subsequent surveillance, and survival.

For the quality assessment, we used the following three items: (1) adequate description of the patients’ demographic data and history; (2) adequate description of the diagnoses (clinical, imaging, histopathologic); and (3) description of the treatment (treatment sequence, results, follow-up).

## 3. Results

### 3.1. Systematic Review of the Literature

We obtained a total of 2162 records, of which 184 were from PubMed, 1750 were from Google Scholar, 197 were from PubMed central, 6 were from Cochrane, and 25 were from the Registries database. We searched in articles, original articles, reviews, case reports, letters to the editor, and clinical trials.

A total of 2162 records were identified in databases. Of these, 197 duplicates were identified and excluded, and 1780 articles were rejected before screening due to their title. The remaining 185 articles were screened, and 27 reports were assessed for eligibility. After applying all exclusion criteria, 17 articles remained.

**Figure 1:**
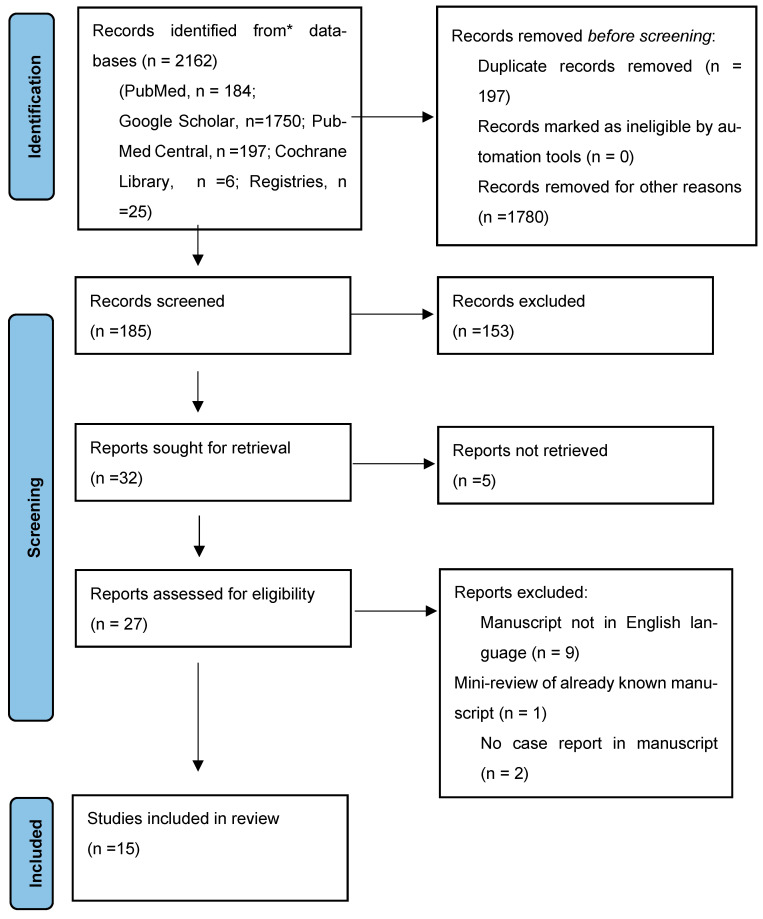
PRISMA flow diagram.

Of these, 2 were original articles and 15 were case reports. One article referred to a case series and was a prognostic analysis, and one article was a mini review of four other articles already included in the study. These two articles were also excluded. A flowchart for the systematic literature review and the articles included in the analysis is shown in Figure 1.

### 3.2. Quality Assessment

The quality of all the included articles was assessed by two researchers (C.R.I., A.G.) who worked independently. The Joanna Briggs Institute (JBI) critical appraisal checklist for case reports was used to establish the risk of bias in the articles that were included. The JBI appraisal checklist for case reports is based on eight questions, each of which uses a score of 1 or 0 (yes = 1, no = 0, and unclear or not applicable = 0). The overall score for each article was presented as a percentage, and the article was characterized according to different degrees of risk of bias (a high risk of bias was assessed if 20–50% of the items scored yes, a moderate risk of bias was assessed if 50–80% of the items scored yes, and a low risk of bias was assessed if 80–100% of the items scored yes, as per the JBI checklist) [22].

The results showed a low risk of bias in eight selected articles and a moderate risk in seven articles (see the appropriate Table S1 in Supplementary Files).

A majority of the articles included provided an appropriate description of the case reports. Some cases did not use TNM staging for diagnosis; however, the data provided in the article were analyzed by all authors to confirm the diagnosis and the stage of neoplastic disease.

### 3.3. General Characteristics

The reviewed articles describe 15 clinical cases of patients diagnosed with synchronous breast and colorectal tumors. We used descriptive statistics to analyze the clinical cases in terms of age, gender, history, diagnosis of the primary tumor, diagnosis of the second tumor, surgical treatment (in an operative or sequential time), adjuvant treatment, and staging of each type of tumor [12,14,23,24,25,26,27,28,29,30,31,32,33,34,35].

For the 15 cases, the gender distribution was as follows: 13 females and 2 males. In terms of age, the two males were 50 and 57 years old, respectively, and the females were between 40 and 79 years old, with a median age of 60 years (Table 1).

In nine cases, the first tumor diagnosed was a colorectal tumor (including two cases with emergency symptoms, e.g., intestinal occlusion), and in six cases, the patients presented with symptoms initially suggestive of a breast tumor (the presence of a palpable breast lump). It is worth mentioning that in a study by Nitipir et al., the presentation to the hospital was also determined by occlusive symptoms, but the patient reported was a pending biopsy for a breast tumor [28].

In about half of the cases, no risk factors were reported; however, the reports were varied. In four cases, data were missing. Only two cases described risk factors possibly associated with the risk of neoplasia (obesity, smoking). The situation is the same for family history; in two cases, results were positive for breast neoplasia; in the rest of the cases, the results were either not reported (five cases) or negative (eight cases).

In the vast majority of cases, the diagnosis of a synchronous tumor was prompted by the first tumor’s workup. In only one case—a patient hospitalized with an intestinal obstruction but waiting for a biopsy of a breast tumor—the two tumors were diagnosed separately (Nitipir et al.) [28]. Obviously, the diagnosis of neoplasia occurred following the biopsies (these included a core biopsy for breast tumors and a biopsy, obtained during colonoscopy, for colorectal tumors).

### 3.4. Breast Tumor Characteristics

Breast tumors had the following three anatomopathological forms: invasive ductal carcinoma (IDC) in 14 cases and invasive lobular carcinoma (ILC) and Paget’s disease in 1 case each. Only one tumor was reported as NST, and this was from an older paper. Due to this paper’s description of the subsequent histological and immunohistochemical results, we classified it as IDC. One case noted the presence of bilateral breast tumors, while eight cases noted left breast tumors, and five cases reported right breast tumors (Table 2).

Regarding staging, there were an equal number of patients in stage I as there were in stage II (five each), and there were three cases with missing information, one case in stage IV, and two cases in stage III.

Concerning immunohistochemical tests, the reports were detailed in cases where hormone receptors were reported (estrogen and progesterone), and the details about the Her status appeared in 12 cases (4 positive and 8 negative). The mention of BRCA testing appeared in only two cases.

### 3.5. Colorectal Tumor Characteristics

Colorectal tumors are exclusively adenocarcinomas.

Localization on the left colon is predominant (eight cases), followed by localization on the right colon (five cases), the rectum (one case), and reports with missing information (one case). In one case, there were two synchronous colorectal tumors (caecum and sigmoid) (Table 3).

Tumor stages were relatively evenly distributed, with five cases each for stage I and III, three cases of stage II, one case of stage IV, and one case with missing information.

### 3.6. Treatment

After performing the investigations and obtaining the biopsy results, a therapeutic decision was reached. All cases had surgical indication (both for breast and colorectal tumor) except one case (I Lin Su et al., 35), in which the breast tumor had metastases at diagnosis (lung and bone tumors) and received adjuvant chemotherapy. In four cases, surgery was performed concomitantly (colectomy and mastectomy) [23,24,30,34]. In three cases, surgery was initially performed for the breast tumor [27,29,31] and was followed by colon surgery at 6-, 14-, and 30-day intervals, respectively (Table 4).

Obviously, in the cases of surgical emergencies (patients presented with bowel obstruction, two cases), surgery was absolutely vital. In another six cases, colonic surgery was indicated first, especially for patients who had undergone chemo-homonotherapy for preoperative breast cancer.

The data in the articles are inconsistent in terms of the exact tumor stage for all tumors diagnosed and in terms of the adjuvant treatment performed (referral to the oncologist).

No estimation can be made about survival because of missing data from the articles.

## 4. Discussion

Although the treatment of respective breast and colorectal cancers is quite standardized, synchronous MPMTs are not included in any oncologic treatment guidelines, and the prognosis is dictated by the most advanced stage of either of the two tumors [32,33].

Firstly, we attempted to analyze the demographic profile of patients with both CRC and breast cancer. The median age in the study was 60. The media reported median age in the literature was 63 for breast cancer and 67 for CRC [1,2,3,4,5,6,7].

We also attempted to analyze risk factors (obesity, alcohol consumption) and family history in the study cases. Unfortunately, there were mentions about risk factors in only two cases, and family history was positive for breast cancer in two cases. For the rest, there were negative records or missing data.

Studies demonstrate that alcohol consumption and obesity increase the risk for both colorectal and breast cancer [36,37,38,39]. Obesity increases the risk of both CRC and breast cancer (in postmenopausal women) but is a protective factor for breast cancer in premenopausal women [40,41,42].

A major challenge is the diagnosis of synchronous MPMTs and how the diagnosis is performed. Most commonly, these cases are diagnosed during the staging of the first tumor diagnosed. Most publications indicate synchronous tumor discovery during breast tumor staging because breast cancer has early signs and symptoms, and the clinical examination is easy to perform (including self-examination) [13]. Testing for a colorectal synchronous tumor (which is most often asymptomatic) is performed with CT, MRI, and PET CT [7,8,9,10,35]. Obviously, a subsequent colonoscopy and biopsy establishes the definite diagnosis.

There are a few cited cases in which breast tumor diagnosis was performed during colorectal tumor staging by the same imaging investigation methods [23]. This was also the situation in our review, where colorectal cancer was the first to be diagnosed in the majority of cases, and breast cancer was the second to be diagnosed. This is partially explained by the fact that colorectal cancer can present complications (such as obstruction); in such cases, the patient requires emergency examination/surgery.

It is worth noting that none of the patients in the study mentioned did not benefit from invasive screening (although more than 75% of them were over 50 years of age, the age at which screening for both breast and colon cancer is mandatory). In the case of men, they did not meet the screening criteria for breast cancer (there is no such thing for men) but could have met the screening criteria for colon cancer (colonoscopy, given their age) [43,44]. There are studies indicating colorectal screening for patients diagnosed with breast cancer [45].

Breast cancer in men is a very rare finding and is not included in any protocol for diagnosis or treatment. The incidence of breast cancer in men is 1 in 100,000, mostly presenting as ER- and PR-positive invasive ductal carcinomas, as in our study. Data regarding coexisting breast and colon cancer in men are scarce [26,46].

Due to the nature of the study (analysis of several articles, without having a unitary reporting profile), there are almost no mentions about genetic testing. In the literature, the presence of BRCA 1 and BRCA 2 mutations is characterized by an increased risk for male and female breast cancer [47]. Hereditary colon cancer is mostly associated with Lynch syndrome [48].

Germline mutation of CHEK2 has been associated with both breast cancer and CRC [49]. In clinical practice, genetic testing should be performed in cases where onset of either disease occurs at a young age.

The hormonal profile of breast tumors was specified in all cases, and the results obtained are similar to those in the literature, in which the incidence of triple-negative breast tumors is low (10–15% of breast cancers) and is possibly related to inherited mutations. It is correlated with a bad prognosis. There was also one case of a BRCA-positive patient with a family history of breast cancer [50].

In the absence of treatment guidelines, the therapeutic approach is varied. The treatment strategy is individualized for each patient (and TB has a key role) and depends on factors such as the stage of each cancer and overall prognosis.

According to some authors, surgical treatment consists, firstly, in the treatment of the tumor with the most advanced TNM stage (and with the worst prognosis), followed by oncological treatment, and then, after some time, treatment for the second tumor is addressed (with or without oncologic therapy, depending on the final staging) [14,23,25,28].

The present study reported more cases of early-stage breast cancer and later stage CRC; therefore, colon cancer was the first to be operated on. We noted three situations when surgery was performed at the same operative time.

An increasing number of authors consider that if a patient has a radical surgical indication for both tumors, then the therapeutic strategy should utilize the following chronology: primary radical surgical approach of both tumors followed by oncological treatment for the tumor in the most advanced stage depending on the final anatomopathology result. Surgery can be performed concomitantly or during the same hospitalization (at an interval of days or weeks) [23,24,30,34,46,48].

Another important dilemma concerns the chemotherapy regimen for these patients, especially when both CRC and breast cancer require one. Finding a blended chemotherapy that addresses both tumors but does not increase toxicity can be challenging [33]. In the clinical cases analyzed, there was missing information in five cases (it is mentioned that the patients were referred to an oncologist). All breast tumors were treated with endocrine therapy (tamoxifen or anastrazole, depending on the patient’s menstrual status) and chemotherapy, depending on the tumor stage. For CRC, in advanced stages, patients received chemotherapy (XELOX, FOLFOX, or FOLFIRI); in two cases, this treatment overlapped with hormonal breast treatment. There is a discussion in the literature regarding whether to discontinue hormonal therapy during chemotherapy for CRC. There are the following two issues here: 5-FU and capecitabine may also act on breast tumors, and tamoxifen in association with chemotherapy for CRC may increase the risk of thrombosis [51,52]. In our cases, there was no mention of stopping hormone therapy. Once again, we emphasize the importance of a multidisciplinary team in the individualized treatment of each case.

Another discussion regarding these patients is about determining the Her status for both breast and stage IV CRC. There are a small number of studies showing the benefit of anti-Her treatment in metastatic CRC. However, if the breast tumor is Her-positive, the treatment administered might also be active for the colon tumor, but there are no guidelines for the treatment of these synchronous tumors [53].

No assessment of survival can be made in the review due to the lack of standardized follow-up data. The only conclusion is that patients had a good immediate outcome. In one case, the patient survived 7 years without signs of neoplastic disease [31]. In four cases, there are mentions of follow-up at 6, 9, 16, and 24 months, respectively [23,25,28,29]. In one case, the patient died at 18 months due to metastatic breast cancer [35].

In terms of CRC, the number of new cases and mortality are generally trending downward, whereas for breast cancer, the number of new cases is increasing and mortality is decreasing [1,2,3,4,5,6,7]. For metastatic breast and colon cancer, the 5-year survival rate is approximately equal at around 30% [54,55,56].

This study has limitations that must be acknowledged. The medical records had a high degree of missing data, particularly for immunochemistry in colorectal cancer and regarding the exact tumor stage and oncologic treatment for both breast cancer and CRC. We also lacked detailed information about personal comorbidities, risk factors, and family history, and we lacked follow-up data. Additionally, there have been significant changes to the standard of care over the years regarding the treatment and investigations of each cancer, which could change management decisions concerning these patients in the future.

## 5. Conclusions

The incidence of MPMT is increasing and, in this sense, more attention should be paid during staging investigations in order to detect, in a timely manner, the presence of another tumor with different localization.

Developing new methods of treatment and investigation may play an important role in the future.

Once again, we emphasize the importance of screening methods, especially since we are discussing diseases with a standardized protocol.

## Figures and Tables

**Table 1:** Authors and demographic parameters.

No	Author/Year	Sex/Age	Primary		Risk Factors	Familial Antecedents
			breast	colon		
1	Karayiannakis A./2011	M/50		+	Obesity	N/A
2	Anania G./2012	F/79		+	N/A	N/A
3	Higgins L./2013	F/67		+	Smoker, cardiovascular disease	Negative
4	Ari A./2016	M/57		+/obstruction	N/A	N/A
5	Yetkin G./2017	F/65		+	Negative	Negative
6	Nitipir C./2018	F/40	+		N/A	N/A
7	Abdulla H./2019	F/58	+		Negative	Negative
8	Abushwemeh M./2021	F/46	+		Negative	Negative
9	Asaad A./2021	F/68	+		Diabetes, cardiovascular disease	Negative
10	Mevlut R.P./2021	F/67	+		N/A	N/A
11	Bin Saleem M.Y./2022	F/50		+	Cardiovascular disease	Negative
12	Alsulaimani A.I./2022	F/46		+/obstruction	Negative	Positive/breast
13	Gadiyaram S./2022	F/57		+	Negative	Negative
14	Baiomy T./2023	F/63		+	Negative	Negative
15	I-Lin Su/2023	F/60	+		Negative	Positive/Breast

**Table 2:** Breast tumor characteristics.

Localization		Histological Type		TNM		Receptor	
Right	5	IDC	14	I	5	Estrogen+	14
Left	8	ILC	1	II	5	Estrogen−	1
Bilateral	1	PAGET	1	III	2	Progesterone+	14
Missing	1			IV	1	Progesterone−	1
				Missing	3	Her+	4
						Her−	8
						BRCA+	0
						BRCA−	1
						Missing	1

**Table 3:** Colorectal tumor characteristics.

Localization		Histological Type		TNM	
Right	5	ADK	16	TIS	1
Left	8			I	5
Rectum	1			II	3
Synchronous left+ right	1			III	5
				IV	1
				Missing	1

**Table 4:** Treatment.

No	TNM CRC	TNM Breast	CRC Surgery	Breast Surgery	Oncologic First		Oncologic Adjuvant	
					CRC	Breast	CRC	Breast
1	I	II	Same time	Same time	-	-	-	+ChT/ET
2	III/I	Missing	Same time	Same time	Missing	Missing	Missing	Missing
3	II	III	1st	2nd	-	+ ET	+	+ChT/ET
4	Missing	Missing	1st/emergency	2nd	-	-	Missing	Missing
5	I	I	2nd	1st	-	-	Missing	Missing
6	III	I	1st/emergency	2nd	-	-	+Cht/RT	+ ET
7	I	III	2nd	1st	-	-	-	+ChT/ET
8	II	II	Same time	Same time	-	-	+	+
9	I	II	1st	2nd	-	-	-	+ ET/RT
10	III	II	2nd	1st	Missing	Missing	Missing	Missing
11	III	II	1st	2nd	-	-	+	+/antiHer
12	II	I/missing	1st	2nd	-	-	+	+ChT/RT/ET
13	IV	I	1st	2nd	-	-	+	+ET
14	III	I	Same time	Same time	-	-	Missing	Missing
15	TIS	IV	1st	No surgery	-	-	-	+

Legend: CRC = colorectal, ET = endocrine therapy, ChT = chemotherapy, RT = radiotherapy.

## Data Availability

Not applicable.

## Supplementary Materials

The following supporting information can be downloaded at: https://www.mdpi.com/article/10.3390/life14081008/s1, Table S1: Risk of bias according to JBI checklist.
