# Supplementary material for: Synchronous Breast and Colorectal Malignant Tumors—A Systematic Review
Source: Life (Basel). 2024 Aug 13;14(8):1008. doi: 10.3390/life14081008 (PMC11355721; doi:10.3390/life14081008)
Supplement: Supplementary file 1 [file life-14-01008-s001.zip › life-3107031-supplementary.pdf]

Author /year	1	2	3	4	5	6	7	8	OVERALL RISK	PERCENTAGE
Karayiannakis A. /2011	1	1	1	1	1	1	0	1	low	87.5%
Anania G./2012	1	0	0	1	1	1	0	1	moderate	62.5%
Higgins L./2013	1	1	1	1	1	1	0	1	low	87.5%
Ari A./2016	0	1	1	1	1	0	0	1	moderate	62.5%
Yetkin G./2017	1	1	1	1	1	1	0	1	low	87.5%
Nitipir C./2018	1	1	1	1	1	1	1	1	low	100%
Abdulla H./2019	1	1	1	1	1	1	1	1	low	100%
Abushwemeh M./ 2021	1	1	1	1	1	0	1	1	low	87.5%
Asaad A./2021	1	1	0	1	1	1	1	1	low	87.5%
Mevlut R.P./2021	0	1	1	1	0	0	0	1	moderate	50%
Bin Saleem M.Y. /2022	1	1	1	1	1	0	0	1	moderate	75%
Alsulaimani A.I. /2022	1	1	1	1	1	0	0	1	moderate	75%
Gadiyaram S./2022	1	0	1	1	1	1	0	1	moderate	75%
Baiomy T./2023	1	1	0	1	0	1	0	1	moderate	62.5%
I-Lin Su/2023	1	0	1	1	1	1	1	1	low	87.5%

Table S1 – Risk of bias according to JBI checklist

Risk of bias legend

1. Were patient’s demographic characteristics clearly described?
2. Was the patient’s history clearly described and presented as a timeline?
3. Was the current clinical condition of the patient on presentation clearly described?
4. Were diagnostic tests or assessment methods and the results clearly described?
5. Was the intervention(s) or treatment procedure(s) clearly described?
6. Was the post-intervention clinical condition clearly described?
7. Were adverse events (harms) or unanticipated events identified and described?
8. Does the case report provide takeaway lessons?
